# Stability of copper acetate at high P-T and the role of organic acids and CO_2_ in metallic mineralization

**DOI:** 10.1038/s41598-020-62250-1

**Published:** 2020-03-25

**Authors:** Zhiyong Ni, Yanjing Chen, Haifei Zheng, Nuo Li, Heping Li

**Affiliations:** 1 State Key Laboratory of Petroleum Resources and Prospecting, China University of Petroleum, Beijing, 102249 China; 2 College of Geosciences, China University of Petroleum, Beijing, 102249 China; 3 Key Laboratory of Orogen and Crustal Evolution, Peking University, Beijing, 100871 China; 4 Xinjiang Research Center for Mineral Resources, Xinjiang Institute of Ecology and Geography, Chinese Academy of Sciences, Urumqi, 830011 China; 5 Institut für Mineralogie, Leibniz Universität Hannover, Hannover, 30167 Germany; 6 Institute of geochemistry, Chinese academy of sciences, Guiyang, 550002 China

**Keywords:** Geology, Geochemistry

## Abstract

Many metal deposits were formed by carbonic fluids (rich in CO_2_) as indicated by fluid inclusions in minerals, but the precise role of CO_2_ in metal mineralization remains unclear. The main components in fluid inclusions, i.e. H_2_O and CO_2_, correspond to the decomposed products of organic acids, which lead us to consider that in the mineralization process the organic acids transport and then discharge metals when they are stable and unstable, respectively. Here we show that the thermal stability of copper acetate solution at 15–350 °C (0.1–830 MPa) provides insight as to the role of organic acids in metal transport. Results show that the copper acetate solution is stable at high *P-T* conditions under low geothermal gradient of <19 °C/km, with an isochore of *P* = 1.89 *T* + 128.58, verifying the possibility of copper transportation as acetate solution. Increasing geothermal gradient leads to thermal dissociation of copper acetate in the way of 4Cu(CH_3_ COO)_2_ + 2H_2_O = 4Cu + 2CO_2_ + 7CH_3_COOH. The experimental results and inferences in this contribution agree well with the frequently observed fluid inclusions and wall-rock alterations of carbonate, sericite and quartz in hydrothermal deposits, and provide a new dimension in the understanding of the role of CO_2_ during mineralization.

## Introduction

Orogenic gold systems are characterized by abundant carbonic fluid inclusions (rich in CO_2_)^1–3^, but the role of CO_2_ in gold mineralization still remains controversial and enigmatic^4–6^. Carbonic fluid inclusions have been recently observed in various types of copper deposits^7–10^ as well as in lode silver, lead-zinc and molybdenum deposits^11,12^. Therefore, there is a need to understand the relationship between CO_2_ and metallic mineralization.

The mutual conversion between CO_2_ and organic matter is common in both nature and human activity, as exemplified by photosynthesis and fossil fuel combustion^13–16^. Organic matter plays a significant role in metal transport and enrichment in low-temperature hydrothermal environments^17^. Carboxylic acids, such as acetum, have been discovered in petroleum brines^18,19^ and fluid inclusions of ore deposits^20^, and have been shown to transport Pb and Zn as complexes at temperature of <250 °C^17,21^. CO_2_ can be transformed into carboxylic by metal catalyst, such as Mn, Pd and Zn^22–24^. This encourages us to infer that, at high *P*-*T* conditions, carboxylic acids and their metallic complexes can be stable and facilitate mobilization, migration and enrichment of ore metals; and then, decompose to CO_2_ with decreasing pressure during upward fluid migration. Thus, the stability of carboxylic acids and their metallic complexes at high *P*-*T* conditions is the key to understand the mechanism of and the role of CO_2_ in mineralization processes, from a new dimension. However, nothing is known about metallic complexes with carboxylic acids at high *P-T* conditions, due to a shortage of experimental data.

**Figure 1:**
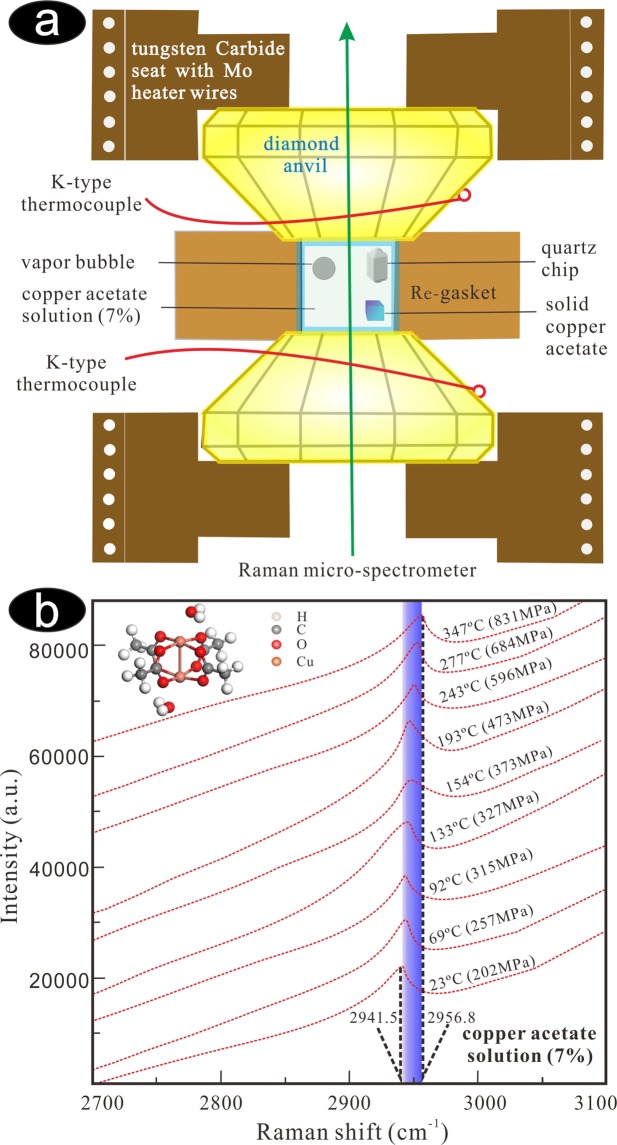
(**a**) The hydrothermal diamond anvil cell (HDAC)^25^. (**b**) The Raman spectra of the C-H symmetry stretching vibration at different temperature and pressure, showing that the copper acetate is stable at high temperature with high pressure (PeakFit V4.12, https://peakfit.updatestar.com).

**Figure 2:**
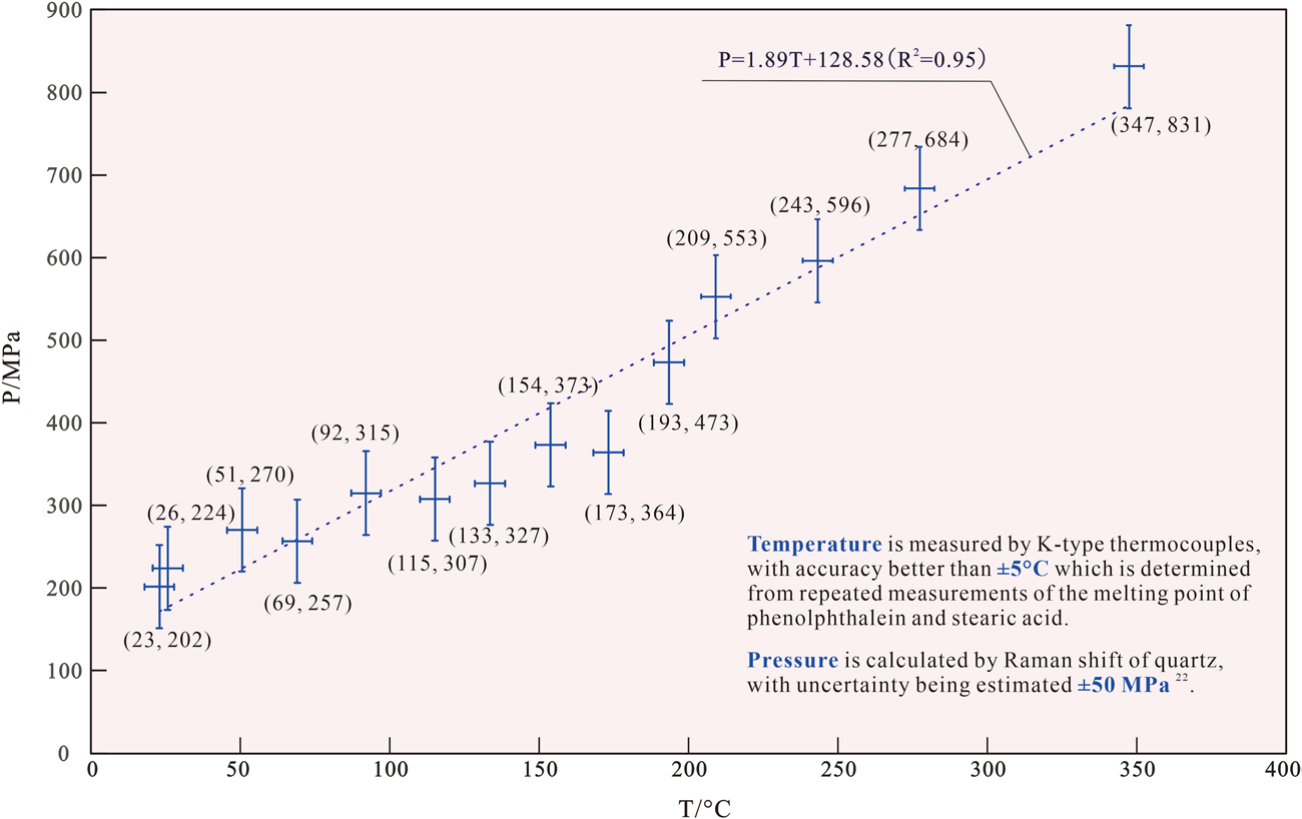
The relation between temperature and pressure in the experiment of copper acetate solution.

To examine the thermal stability of metallic complexes with carboxylic acids at high *P-T* conditions, we have conducted experiments on copper acetate solution (7%), using a diamond anvil cell. Despite of strong fluorescence impact of diamond, the symmetry stretching vibration of C-H bond (about 2,941 cm^−1^), i.e. (υP)_2941_, was observed in copper acetate solution (Fig. 1, Table 1). In the heating process, the shape of the spectra of the copper acetate solution did not change (Fig. 1), and no new peak appeared on the Raman spectra. The volume of the copper acetate solution is constant and the system evolves along the isochore. In other words, system pressure increases with increasing temperature. This is consistent with the relationship between the Raman shift of quartz (464 cm^−1^) and pressure (Fig. 2, Table 1). Thus, the isochore of the copper acetate solution is defined as *P* = 1.89 *T* + 128.58 (Fig. 2), and equals to a geothermal gradient of 19 °C/km. This indicates that copper acetate is stable at temperatures up to 350 °C under low geothermal gradient conditions.

**Table 1:** The Raman shift of quartz and the symmetry stretching vibration of C-H.

Copper acetate solution in diamond anvil cell				Copper acetate solution in moissanite anvil cell			
*T*/°C	*P*/MPa	ν_464_/cm^−1^	ν_2941_/cm^−1^	*T*/°C	*P*/MPa	ν_464_/cm^−1^	ν_2941_/cm^−1^
23	202	465.8	2941.5	16	522	468.7	2949.0
26	224	465.9	2942.7	32	557	468.8	2949.6
51	270	466.0	2941.3	49	603	469.0	2949.9
69	257	465.7	2944.0	65	611	468.8	2950.1
92	315	465.8	2943.7	87	367	466.4	2948.0
115	307	465.4	2942.9	107	355	466.0	2946.7
133	327	465.3	2944.4	120	417	466.3	2947.1
154	373	465.4	2946.9	133	369	465.7	2946.9
173	364	465.0	2947.2	150	478	466.4	2947.6
193	473	465.7	2945.2	156	476	466.3	2948.3
209	553	466.1	2946.5	164	497	466.4	2948.5
243	596	465.9	2951.7	185	512	466.2	2948.0
277	684	466.1	2952.6	197	595	466.7	2948.8
347	831	466.3	2956.8	212	512	465.7	2948.9

P: Pressure; ν2941: Raman shift of the symmetry stretching vibration of C-H; T: Temperature; ν464: Raman shift of the quartz.

**Figure 3:**
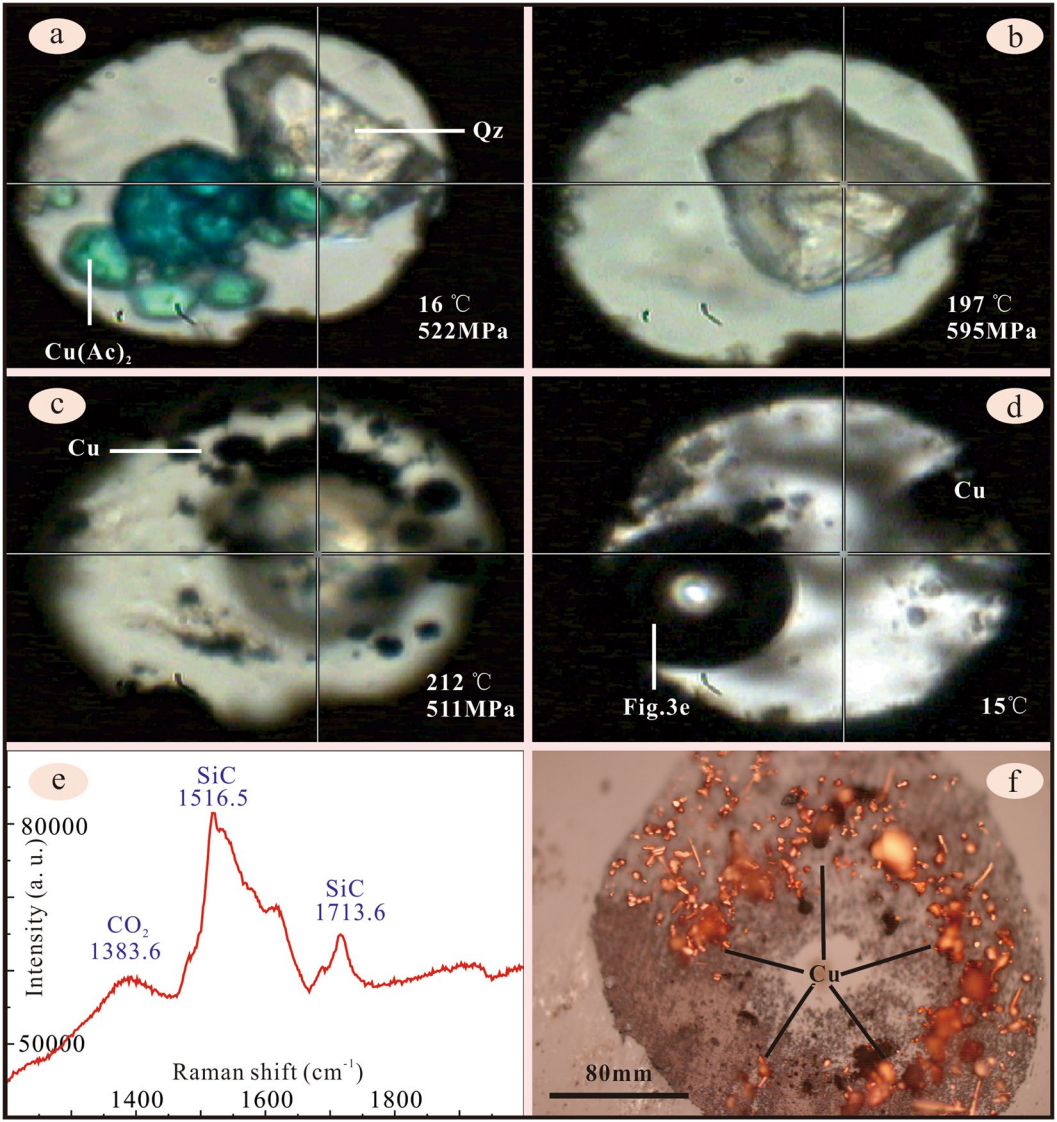
The experiment was conducted in a moissanite anvil cell. The solid copper acetate (Cu(Ac)_2_), quartz (Qz) and copper acetate solution (7%) were enclosed in the hole of a thin rhenium metal gasket (**a**) With the increment of temperature and pressure, the solid copper acetate dissolved to form blue solution. (**b**) At 212 °C/ 511 MPa, black grains appeared (**c**), which were identified to be native copper under microscope (**f**). When the temperature reduced to 15 °C, the vapour bubble appeared (**d**), which was proven to be CO_2_ by Laser Raman (**e**). Unfortunately, we failed to get the pressure at 15 °C due to coating of native copper on the quartz grain.

**Figure 4:**
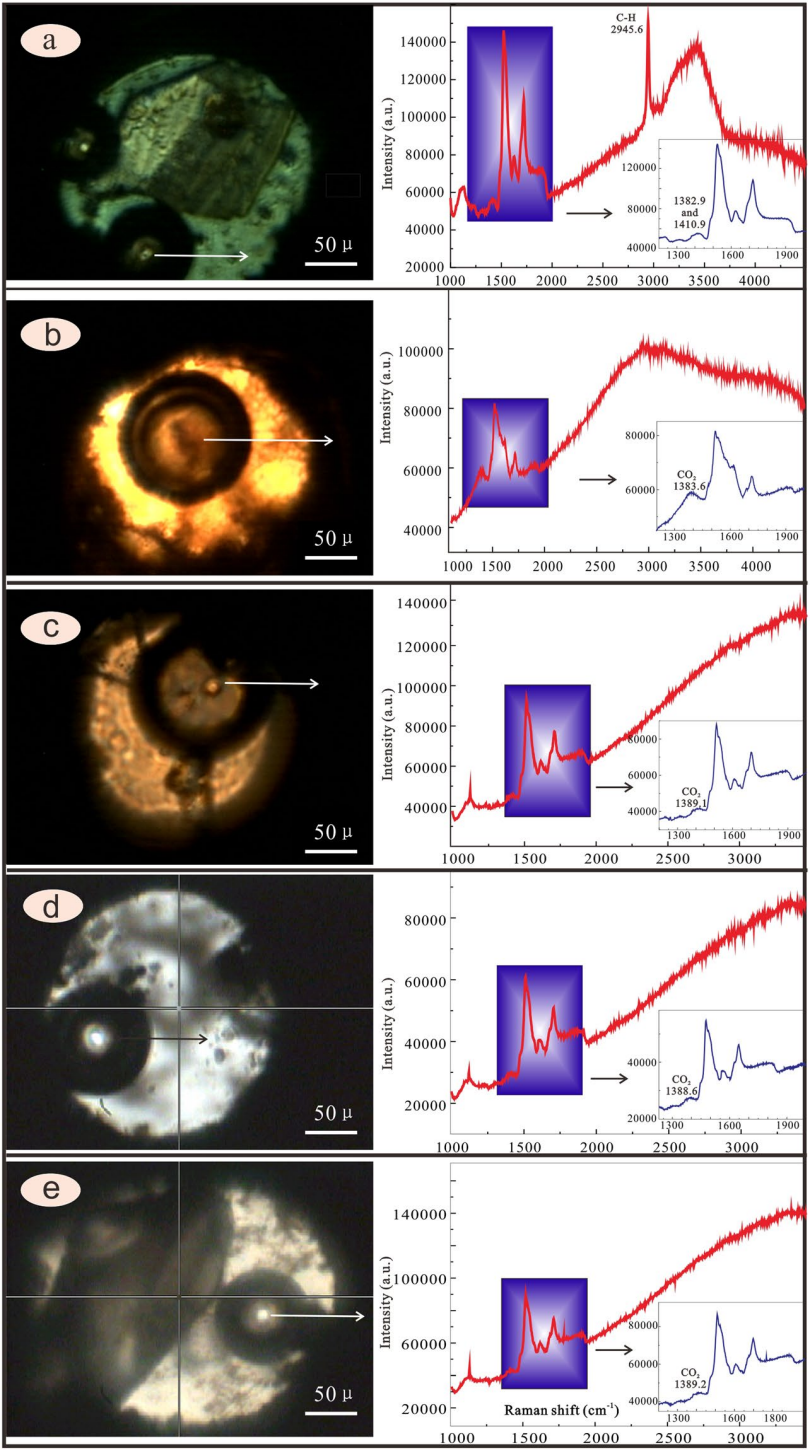
Laser Raman spectra of the vapour and solution. (**a**) The C-H symmetry stretching vibration is obvious (2945.6), illustrating the copper acetate still exist. The two peaks can be observed (1382.9 and 1410.9), is the peak of CO_2_ and C=O of Cu(Ac)_2_, respectively. (**b**–**e**) The C-H symmetry stretching vibration is unobvious or vanishing, illustrating the copper acetate does not exist almost. The unique peak can be observed (1383.6, 1389.1, 1388.6 and 1389.2), showing the peak of CO_2_ (PeakFit V4.12, https://peakfit.updatestar.com).

The thermal dissociation experiment of copper acetate was conducted with a moissanite anvil cell to avoid strong fluorescence. The sample chamber was filled with copper acetate solution (7%), solid copper acetate and quartz chip (Fig. 3a). The chamber was heated step-by-step from 16 °C to 212 °C, with step interval of 6–22 °C, heating rate of 2 – 5 °C/min and pressure ranging 355–611 MPa (Table 1). Each step lasted for 10–15 minutes to achieve stable temperature and pressure, and to acquire the Raman shift of copper acetate solution. The peak symmetry stretching vibration of C-H bond (about 2,941 cm^−1^) shifts to higher frequency along with increasing temperature and pressure (Table 1). During heating, the solid copper acetate firstly dissolved (Fig. 3b), and then vapour bubble (Fig. 3d) and native copper grains (Fig. 3c,d,f) appeared. Under microscope, it was observed that solid copper grains suddenly formed at the conditions of 212 °C and 511 MPa, and the experiment stopped if no more copper precipitated. The vapour bubble was composed of CO_2_, as indicated by the Raman shift (Figs. 3e, 4). Thus, it is concluded that the copper acetate solution is stable at high *P-T* conditions under low geothermal gradient, and thermally dissociated when the geothermal gradient increases, in the way as below:

1 $$4{{\rm{Cu}}({\rm{CH}}}_{3}{{\rm{COO}})}_{2}+2{{\rm{H}}}_{2}{\rm{O}}=4{\rm{Cu}}+{{\rm{2CO}}}_{2}+7{{\rm{CH}}}_{3}{\rm{COOH}}$$

From the reaction Eq. 1 and experiment, new understandings can be drawn out: (1) the organic acids can facilitate metallic transportation via fluids during hydrothermal mineralization. (2) CO_2_ serves as an important buffer to maintain metallic transportation^3,5^, because the existence of CO_2_ in fluid makes the reaction 1 proceeds to the left, keeping CH_3_COO^−^ stable. (3) The copper acetate solution is stable under high-pressure, and therefore, decompression causes copper acetate dissociation, CO_2_ escape and Cu precipitation, as similar to those revealed in previous studies^1–12^. (4) Wall-rock carbonation removes CO_2_ from the solution, and results in precipitation of metals. (5) Decreasing *p*H can facilitate copper acetate stability and transportation; by contrast, increasing *p*H accelerates copper acetate dissociation and Cu precipitation, and also causes phyllic alteration (sericite + quartz) in the way of Eq. 2:

2 $$3{{\rm{KAlSi}}}_{3}{{\rm{O}}}_{8}+2{{\rm{CH}}}_{3}{\rm{COOH}}={{\rm{KAl}}}_{2}[{{\rm{AlSi}}}_{3}{{\rm{O}}}_{10}]{({\rm{OH}})}_{2}+6{{\rm{SiO}}}_{2}+2{{\rm{KCH}}}_{3}{\rm{COO}}$$

Therefore, the common observation of carbonate, sericite and quartz alterations, and CO_2_-rich fluid inclusions in hydrothermal deposits^11^, such as the orogenic-type Cu lodes, corresponds well with the experimental results of stability and thermal dissociation of copper acetate solution.

## Methods

The experiment was performed in hydrothermal diamond and moissanite anvil cells^25,26^, respectively. The sample was enclosed in the 200–400 μm diameter hole of a thin (300–400 μm) rhenium metal gasket by compressing the gasket between two diamond anvil faces^27^. The temperature of the diamond anvils and samples was controlled and measured using Mo resistance heaters and two attached K-type thermocouples, respectively^27^. Temperature measurement was corrected using the melting point of phenolphthalein and stearic acid, and the accuracy of reported temperatures is within ±5 °C. A small chip of quartz (0.18–0.20 mm) was put in cell to calibrate internal pressure. Experimental pressure was determined according to the relationship between the Raman shift of quartz and the pressure^28,29^_._

Raman spectroscopy was performed using a Raman micro-spectrometer (Renishaw system RM-1000, Renishaw Group, Gloucestershire, United Kingdom); the slit width was set at 50 μm and the resulting resolution was ±1 cm^−1^ ^30^. The objective is a Leitz 20× with a working distance of 15 mm. An argon ion laser with a wavelength of 514.5 nm operated at 20 mW was used to illuminate the sample for Raman signal generation. Each spectrum was collected within an accumulation time of 30 s and covering a wavelength of 100–4,000 cm^−1^ ^30^. The initial experimental temperature was 15 °C, which was gradually increased to 350 °C. In the experiment, the Raman spectrum test was conducted 3–5 min after each change in experimental temperature to ensure that the samples firstly reach equilibrium. The results were processed using PeakFit software.

## References

[1] Goldfarb RJ (2005). Distribution, character and genesis of gold deposits in metamorphic terranes. Economic Geology.

[2] Kerrich R, Goldfarb RJ, Groves DI, Garwin S, Jia YF (2000). The characteristics, origins, and geodynamic settings of supergiant gold metallogenic provinces. Science in China Series D.

[3] Pirajno, F. *Hydrothermal processes and mineral systems*. (Springer, 2009).

[4] Heinrich CA (2007). Fluid-fluid interactions in magmatic-hydrothermal ore formation. Reviews in Mineralogy and Geochemistry.

[5] Liebscher A (2007). Experimental studies in model fluid systems. Reviews in Mineralogy and Geochemistry.

[6] Phillips GN, Evans KA (2004). Role of CO_2_ in the formation of gold deposits. Nature.

[7] Cailteux JLH, Kampunzu AB, Lerouge C, Kaputo AK, Milesi JP (2005). Genesis of sediment-hosted stratiform copper-cobalt deposits, central African Copperbelt. Journal of African Earth Sciences.

[8] Li WB, Lai Y, Sun XW, Wang Bao Guo (2007). . Fluid inclusion study of the Bainaimiao Cu-Au deposit in Inner Mongofia, China. Acta Petrologic Sinica.

[9] Richards JP, Krogh TE, Spooner ETC (1988). Fluid inclusion characteristics and U-Pb rutile age of late hydrothermal alteration and veining at the Musoshi stratiform copper deposit, Central African copper belt, Zaire. Economic Geology.

[10] Wu KW, Zhong H, Zhu WG, Leng CB, Gou TZ (2008). Study on ore forming fluid of the Dahongshan stratiform copper deposit, Yunnan, China. Acta Petrologic Sinica.

[11] Chen YJ (2007). Diagnostic fluid inclusions of diferent types hydrothermal gold deposits. Acta Petrologic Sinica.

[12] Ni ZY, Li N, Guan SJ, Zhang H, Xue LW (2008). Characteristics of fluid inclusions and ore genesis of the Dahu Au-Mo deposit in the Xiaoqinling gold field, Henan province. Acta Petrologic Sinica.

[13] Jin FM (2011). High-yield reduction of carbon dioxide into formic acid by zero-valent metal/metal oxide redox cycles. Energy & Environmental Science.

[14] Jin FM, Zeng X, Jing ZZ, Enomoto H (2012). A potentially useful technology by mimicking nature—rapid conversion of biomass and CO_2_ into chemicals and fuels under hydrothermal conditions. Industrial & Engineering Chemistry Research.

[15] Michiels K, Peeraer B, Van Dun W, Spooren J, Meynen V (2015). Hydrothermal conversion of carbon dioxide into formate with the aid of zerovalent iron: the potential of a two-step approach. Faraday Discussions.

[16] Zhong H (2019). Selective conversion of carbon dioxide into methane with a 98% yield on an *in situ* formed Ni nanoparticle catalyst in water. Chemical Engineering Journal.

[17] Giordano, T. H. In *Organic acid**s in**geological processes* (eds. Pittman, E. D. & Lewan, M. D.) 319–355 (Springer, 1994).

[18] Bell JLS, Palmer DA (1994). Thermal decomposition of acetate: III. Catalysis by mineral surfaces. Geochimica et Cosmochimica Acta.

[19] Fisher JB, Boles JR (1990). Water-rock interaction in Tertiary sandstones, San Joaquin Basin, California, USA: diagenetic controls on water composition. Chemical Geology.

[20] Sun Q, Zeng YS (1998). Carboxylate composition of fluid inclusion leachate in the Linglong gold deposit, Shandong province, China. Geochemica.

[21] Lewan, M. D. & Pittman, E. D. In *Organic Acids in Geological Processes* (eds. Pittman, E. D. & Lewan, M. D.) 319–355 (Springer, 1994).

[22] Jin F (2014). Highly efficient and autocatalytic H_2_O dissociation for CO_2_ reduction into formic acid with zinc. Scientific Reports.

[23] Lyu LY, Zeng X, Yun J, Wei F, Jin FM (2014). No catalyst sddition and highly efficient dissociation of H_2_O for the reduction of CO_2_ to formic acid with Mn. Environmental Science & Technology.

[24] Zhong H, Yao HS, Duo J, Yao GD, Jin FM (2016). Pd/C-catalyzed reduction of NaHCO_3_ into CH_3_COOH with water as a hydrogen source. Catalysis Today.

[25] Bassett, W. A., Shen, A. H., Bucknum, M. & Chou, I. M. A new diamond anvil cell for hydrothermal studies to 10 GPa and from 190 °C to 1100 °C. *Review of Scientific Instruments* **64**, 2340–2345 (1993).

[26] Xu JA, Mao HK (2000). Moissanite: A window for high-pressure experiments. Science.

[27] Chou IM, Anderson AJ (2009). Diamond dissolution and the production of methane and other carbon-bearing species in hydrothermal diamond-anvil cells. Geochimica et Cosmochimica Acta.

[28] Sun Q, Wang QQ, Ding DY (2014). Hydrogen bonded networks in supercritical water. The Journal of Physical Chemistry B.

[29] Schmidt C, Ziemann MA (2000). *In-situ* Raman spectroscopy of quartz: A pressure sensor for hydrothermal diamond-anvil cell experiments at elevated temperatures. American Mineralogist.

[30] Qiao EW, Zheng HF, Xu B (2009). Raman scattering spectroscopy of phase transition in n-pentadecane under high temperature and high pressure. Chinese Physics Letters.

